# Histological antiphospholipid-associated nephropathy versus lupus nephritis in patients with systemic lupus erythematosus: an observational cross-sectional study with longitudinal follow-up

**DOI:** 10.1186/s13075-015-0614-5

**Published:** 2015-04-27

**Authors:** Jakob Gerhardsson, Birgitta Sundelin, Agneta Zickert, Leonid Padyukov, Elisabet Svenungsson, Iva Gunnarsson

**Affiliations:** Department of Medicine, Unit of Rheumatology, Karolinska University Hospital, Karolinska Institutet, S-171 76 Stockholm, Sweden; Department of Pathology and Cytology, Karolinska University Hospital, Karolinska Institutet, S-171 76 Stockholm, Sweden

## Abstract

**Introduction:**

Renal involvement is a severe complication in systemic lupus erythematosus (SLE). Moreover, a subset of SLE patients develop the anti-phospholipid syndrome (APS), characterised by the occurrence of anti-phospholipid antibodies in combination with macro- and microvascular thrombotic manifestations, including acute and chronic antiphospholipid-associated nephropathy (APLN). Clinical presentations of lupus nephritis and APLN are similar and a renal biopsy is necessary to differentiate between the conditions. Our aim with this study was to investigate the occurrence of histopathological findings consistent with APLN (hAPLN) in renal biopsies from SLE patients and to investigate associations with anti-phospholipid antibody specificities, clinical manifestations, HLA-DRB1 alleles, and long-term renal outcome.

**Method:**

Consecutive renal biopsies from 112 SLE patients with renal involvement were investigated and evaluated for findings of hAPLN; in all there were 236 renal biopsies. Data from biopsy reports and clinical information were collected. Autoantibodies against cardiolipin and β_2_-glycoprotein-1 were measured by enzyme-linked immunosorbent assay. A lupus anticoagulant test was determined with a modified Dilute Russel Viper Venom method. HLA genotyping was performed by sequence-specific primer PCR. Renal outcome was determined at study end.

**Results:**

The prevalence of hAPLN was 14.3% among SLE patients with renal involvement. Compared to patients with pure lupus nephritis, occurrence of hAPLN was associated with intima changes (odds ratio (OR) = 24; 95% confidence interval (CI), 3.0 to 189.8; *P* < 0.0001), hypertensive vascular changes (OR = 7.8; 95% CI, 1.6 to 39.4; *P* = 0.01), inflammatory infiltrates (OR = 6.5; 95% CI, 1.7 to 25.1; *P* = 0.007) and tubular atrophy (OR = 13.1; 95% CI, 1.7 to 103.6; *P* = 0.002). hAPLN was associated with the presence of cardiolipin antibodies (OR = 3.3; 95% CI, 1.0 to 10.8; *P* = 0.05) and triple anti-phospholipid antibody positivity (OR = 4.2; 95% CI, 1.3 to 13.7; *P* = 0.02). Patients with hAPLN were more hypertensive (OR = 3.8; 95% CI, 1.2 to 12.3; *P* = 0.03) and had higher levels of creatinine as compared to lupus nephritis patients (median 116 versus 75 μmol/L; *P* < 0.0001). We found significantly higher frequency of HLA-DRB1*13 (OR = 5.1; 95% CI, 1.7 to 15.4; *P* = 0.03) and development of end-stage renal disease (OR = 5.8; 95% CI, 1.7 to 19.7; *P* = 0.008) in hAPLN compared with lupus nephritis.

**Conclusion:**

hAPLN is a severe and often unrecognized condition in SLE patients with renal involvement. We have demonstrated an increased risk for development of renal impairment and a genetic predisposition in hAPLN patients compared to lupus nephritis patients.

## Introduction

Systemic lupus erythematosus (SLE) is a systemic autoimmune disease characterized by a heterogeneous set of clinical presentations including renal manifestations and the presence of autoantibodies. Antiphospholipid antibodies (aPL) are detected in 30 to 50% of patients with SLE and are associated with thromboembolic complications. Lupus nephritis (LN) of varying severity affects up to 60% of SLE patients [1,2]. Apart from the glomerular lesions seen in LN, patients with SLE may develop renal microangiopathy which may be identical to findings seen in patients with the antiphospholipid syndrome (APS) [3]. The microangiopathy can be acute or chronic and may occur in isolation or with concurrent LN [4-7]. Of note, the clinical presentations are similar and a renal biopsy is required to distinguish APS-related findings from pure LN.

In the most recent consensus criteria of APS [3], the term antiphospholipid-associated nephropathy (APLN) was suggested to describe the entity of aPL in association with renal vasculopathy. In previous publications, however, various definitions have been used to describe the acute condition thrombotic microangiopathy (TMA) [7], isolated renal microthrombi [5], or both acute and chronic renal findings [4,6]. Thus, full comparisons between studies on renal vasculopathy in SLE populations are difficult to perform. It is also of note that aPL have not been identified in all cases with APLN in SLE [4,7] and the natural history of APLN development over time is largely unknown.

aPL are a family of autoantibodies which target membrane structures such as phospholipids or phospholipid-binding proteins. In clinical practice, aPL positivity is determined by occurrence of anticardiolipin antibodies (aCL) or of those to the protein co-factor β_2_-glycoprotein-1 (β_2_GP1) as measured by enzyme-linked immunosorbent assay (ELISA), or positivity in the functional lupus anticoagulant (LA) test. LA detects aPL, which are capable of prolonging *in vitro* coagulation assays [3,8,9], and has been shown to be more closely related to clinical symptoms than positive ELISA tests [1,10]. With regard to APLN, data have been divergent concerning the associations with different aPL [4-7].

There is a genetic predisposition to certain sub-phenotypes of SLE, including nephritis [11,12]. Furthermore, variations in human leukocyte antigen (HLA) genes have previously been found to associate with aPL in SLE [13]. We have recently demonstrated an association between the development of both aPL and macrovascular thrombotic events among SLE patients with the HLA-DRB1 alleles and with the polymorphism in the signal transducer and activator of transcription factor 4 (*STAT4*) gene [12,14]. The *STAT4* risk allele on chromosome 2 was also recently demonstrated to be associated with LN as well as with the development of severe renal insufficiency, thus further indicating that genetic predisposition is of importance for disease progression in LN [11]. No genetic studies on APLN were known at the time of our investigation.

We have investigated the occurrence of histopathological renal microangiopathy in accordance with APLN in consecutive biopsies from a large cohort of SLE patients with renal involvement. We chose to define the renal vascular findings, regardless of antibody findings, as histopathological APLN (hAPLN). We studied the renal outcome in patients with hAPLN in comparison to patients with pure LN and investigated the occurrence of hAPLN over time in repeated renal biopsies. Although hAPLN has been associated with more severe renal disease in SLE [4-6], data on long-term outcome are still an issue of concern and were therefore investigated in this study. Finally, we studied the impact of HLA-DRB1 alleles on the occurrence of hAPLN.

## Methods

### Patients

Consecutive renal biopsies performed between 1995 and 2011 in patients included in the Karolinska SLE Cohort [12] were used in this study. All patients fulfilled at least four classification criteria for SLE [15] and were recruited from both the rheumatology and nephrology units in order to obtain a representative selection with regard to renal involvement. Information was collected from first study biopsy until 31 December 2012. Patients with renal findings that were not related to LN or hAPLN were not included in the study. All patients had given informed written consent to participate in the study and the study protocol was reviewed and approved by the regional ethics committee at Karolinska Institutet in Stockholm, Sweden.

### Renal biopsies and clinical information

All renal biopsies were evaluated for findings in accordance with hAPLN and/or LN. Simultaneously with the biopsies, clinical and laboratory data including occurrence of hypertension, treatment with corticosteroids, anticoagulants, and antihypertensive medication were collected.

Hypertension was considered as present if patients were diagnosed with and prescribed medications for high blood pressure or if the patient had a blood pressure >140/90 mmHg at time of biopsy. Patients treated with angiotensin converting enzyme inhibitors and/or angiotensin receptor blockers for anti-proteinuric indications only were not regarded as hypertensive.

### Evaluation of renal biopsies

All biopsies were evaluated by one experienced pathologist (BS) and examined with light microscopy, immunofluorescence and electron microscopy. Glomerular lesions were assessed according to World Health Organization criteria [16] and/or the International Society of Nephrology/Renal Pathology Society criteria [17] depending on the date of biopsy. Information about vascular findings, intima change, interstitial fibrosis, inflammatory infiltrates, tubular atrophy and hypertonic vascular changes as well as findings from electron microscopy were also extracted from the pathology reports.

Vascular findings consistent with APLN [3] were reported and further divided into acute TMA and chronic hAPLN. TMA was defined as occurrence of fibrin thrombi in arterioles and/or glomerular capillaries. Definition of chronic features of hAPLN included presence of fibrous intimal hyperplasia with or without recanalization, fibrous and/or fibrocellular occlusions of arteries and arterioles and focal cortical atrophy [4,6,18]. In cases where acute TMA occurred without concomitant LN, other conditions that may cause endothelial dysfunction were excluded before inclusion in the study. Cases in which a definite diagnosis of hAPLN was doubtful were re-assessed by the pathologist for a definite diagnosis.

### Laboratory data

Laboratory data were analysed at the time of the biopsy and performed according to clinical routine. In this study we included levels of creatinine (μmol/L), albumin (g/L) and haemoglobin (g/L).

### Autoantibodies

Autoantibodies were analyzed in the Department of Clinical Immunology at the Karolinska University Hospital. For aCL and anti-β_2_GP1 antibody detection, ELISA and multiplex methods were used. aCL was evaluated for both immunoglobulin (Ig)G and IgM isotypes. For anti-β_2_GP1, only the IgG isotype was measured. We also recorded if patients had a positive LA, determined with a modified Dilute Russel Viper Venom method (Biopool, Umeå, Sweden).

Being a retrospective observational study (spanning January 1995 to December 2012), methods for autoantibody detection have changed during the study period. Tabulated data were thus recorded as positive or negative according to the cut-off levels of the routine method used at the time of analysis.

The data available in the Karolinska SLE Cohort included current and retrospective information regarding serological profile and occurrence of aCL and anti-β_2_GP-1, as well as LA positivity. Autoantibody and LA positivity was also determined at the time of renal biopsy. Autoantibody and/or LA positivity was thus determined both at and before biopsy, which enables a confirmation of autoantibody findings as required to fulfil the classification criteria for definite APS according to Miyakis and colleagues [3]. Furthermore, we determined if the patients were ever positive in all three aPL tests (aCL (IgM and IgG), anti-β2gGP-1 (IgG) and LA). This combination is described by Pengo and colleagues as triple positivity [19], and is associated with a more severe prognosis.

### Long-term follow-up

The renal function was estimated at last follow-up by the Modified Diet in Renal Disease formula [20], and the patients were classified into chronic kidney disease classes 1 to 5 [21]. We defined the time the patient reached chronic kidney disease class 5 as end-stage renal disease (ESRD).

### HLA typing

Two-digit HLA-DRB1 genotyping for the HLA-DRB1-gene was previously performed by sequence-specific primer PCR and was also used for the present study [12]. We studied the following HLA-DRB1 allelic groups: DRB1*01, DRB1*03, DRB1*04, DRB1*07, DRB1*08, DRB1*09, DRB1*10, DRB1*11, DRB1*12, DRB1*13, DRB1*14, DRB1*15 and DRB1*16 using dominant model.

### Statistics

Chi-squared test and Fisher’s exact test were used as appropriate for statistical analysis of selected categorical variables, which included vascular changes consistent with hAPLN, presence of aCL, anti-β_2_GP1 autoantibodies, positive tests for LA, and hypertension. Odds ratios (ORs) were calculated from contingency tables assessing the risk of co-appearance of selected variables. For variables with statistical significance, 95% confidence intervals (CIs) were calculated.

Skewed continuous variables were log transformed to obtain a normal distribution. For quantitative variables, such as creatinine, albumin and haemoglobin, analysis of variance was used. Mean and extremes are given for descriptive purposes. Bonferroni correction was performed for multiple comparisons when tabulating HLA genotypes.

For statistical analysis JMP-11 software (SAS institute, NC, USA) was used. Statistical significance was accepted at *P* < 0.05.

## Results

### General patient characteristics

The original study cohort consisted of 126 patients with a diagnosis of SLE who had renal biopsy performed due to signs of renal involvement. Fourteen patients were excluded from the study due to histopathological findings other than LN or hAPLN (seven with focal necrotizing vasculitis in the active or inactive phase, one with IgA nephropathy, one with thin basal membranes, one with tubulointerstitial nephritis in accordance with Sjögren’s syndrome, and four with unspecific chronic lesions with no prior history/biopsy with LN). The final study cohort thus consisted of 112 patients who had been subject to 236 biopsies in total. The median number of biopsies per patient was 2 (range 1 to 7) and the median age at first study biopsy was 38 years (range 18 to 84 years).

There were 89 females (79.5%) and 23 males (20.5%). In all, 201 biopsies (85.2%) were from female patients and 35 (14.8%) from male participants. Patient characteristics are displayed in Table 1.

**Table 1:** **Clinical characteristics in all patients at first study biopsy**

	**All patients**	**LN**	hAPLN*	***P*** **value**
	**(n = 112)**	**(n = 96)**	**(n = 16)**	
Median age (years (range))	38 (18-84)	37 (18-84)	45 (26-62)	0.37
Male/female	23/89	18/78	5/11	0.3
Serum creatinine (μmol/L; median)	77	75	116	**<0.0001**
Serum albumin (g/L; median)	29	29	30	0.9
Haemoglobin (g/L; median)	113	114	110	0.2
Hypertension (n (%))	41 (37)	30 (31)	11 (69)	**0.03**
Systolic blood pressure (mmHg; median)	125	120	137	**0.01**
Diastolic blood pressure (mmHg; median)	80	80	80	0.1
Autoantibodies (n (%))*				
aCL	58 (52)	46 (48)	12 (75)	**0.05**
anti-β_2_GP1	23 (21)	17 (18)	6 (38)	0.07
LA	28 (25)	21 (22)	7 (44)	0.06
Single positive aPL	36 (32)	31 (32)	5 (31)	0.9
Double positive aPL	9 (8)	8 (8)	1 (6)	0.8
Triple positive aPL	18 (16)	12 (13)	6 (38)	**0.02**
Definite APS	13 (12)	10 (10)	3 (19)	0.3
Treatment (n (%))				
Prednisolone (mg/day; median (range))	8 (0-60)	8 (0-40)	15 (0-60)	**0.006**
ACEi/ARB	40 (36)	29 (30)	11 (69)	**0.007**
Warfarin	10 (9)	7 (7)	3 (19)	0.2
ASA	16 (14)	13 (14)	3 (19)	0.7

*Previous findings and/or at renal biopsy. *P* values are the comparison between the two diagnostic groups (LN/hAPLN). Values in bold are significant at <0.05. Single positive, double positive and triple positive refers to autoantibody findings at or before biopsy. ACEi, angiotensin converting enzyme inhibitor; aCL, anticardiopin antibodies; anti-β_2_GP1, anti-β_2_-glycoprotein-1; APS, antiphospholipid syndrome; ARB, angiotensin receptor blocker; ASA, acetylsalicylic acid; hAPLN, histopathological antiphospholipid-associated nephropathy; LA, lupus anticoagulant; LN, lupus nephritis.

### Renal biopsies

Overall, 16/112 patients (14.3%) had findings in accordance with hAPLN in at least one biopsy. Among all biopsies, hAPLN was detected in 20/236 (8.5%). Thus, four patients had two biopsies with hAPLN findings. Ten biopsies revealed findings of LN with concomitant findings of hAPLN while 10 had pure hAPLN. Of 236 biopsies, 226 (95.8%) had changes compatible with LN according to either the World Health Organization [16] or International Society of Nephrology/Renal Pathology Society criteria [17].

Of the sixteen hAPLN patients, seven (43.8%) had findings in their first study biopsy while 9/16 (56.2%) patients developed hAPLN following an initial diagnosis of LN (median 8.3 years, range 1 to 28 years) from first biopsy. Conversely, in three cases hAPLN was seen in the first study biopsy, but not in a repeated biopsy. In the hAPLN group, 4/20 (20%) biopsies were consistent with the acute setting (TMA), while the majority 16/20 (80%) had a more chronic phase of hAPLN.

For statistical purposes, when comparing clinical/laboratory variables in the LN group (n = 96) versus the hAPLN group (n = 16), we used data collected at the first study biopsy in the LN patients and the first biopsy demonstrating hAPLN findings in the hAPLN group. Thus, nine patients with initial LN were included in the LN group and data from the first hAPLN biopsy was used for comparison between the groups. No association between age or gender comparing the LN and hAPLN groups was found. Data on all biopsy findings in the hAPLN patients are shown in Table 2.

**Table 2:** Histopathological classification*, age and development of end-stage renal disease in hAPLN patients

**Patient number**	**Biopsies prior to study start (age)**	**Biopsy prior to hAPLN (age)**	**Biopsy I (age)**	**Biopsy II (age)**	**Biopsy III (age)**	**ESRD (age)**
**1**	-	-	hAPLN (54)	-	-	-
**2**	II (36)	-	hAPLN/IV (38)	II (39)	V (41)	+ (55)
**3**	-	-	hAPLN (49)	hAPLN (53)	-	-
**4**	hAPLN (8)	-	hAPLN/II (28)	-	-	+ (28)
**5**	-	IV (35)	hAPLN (47)	-	-	-
		II (36)				
		IV (39)				
		IV(42)				
**6**	IV (26)	V (35)	hAPLN/V (36)	-	-	-
	IV (28)					
	III (29)					
**7**	-	II (40)	hAPLN/V (43)	-	-	-
**8**	-	II (27)	hAPLN (29)	Scarring (29)	hAPLN (34)	+ (38)
**9**	-	III (25)	hAPLN/II (26)	-	-	-
**10**	V (16)	V (22)	hAPLN/V (27)	V (33)	-	-
		V (23)				
		V (24)				
		V (26)				
**11**	II + V (24)	-	hAPLN/II + V (52)	-	-	-
**12**	-	-	hAPLN/V (30)	hAPLN/V (33)	-	+ (36)
**13**	-	-	hAPLN/V (58)	V (59)	-	-
**14**	-	-	hAPLN (61)	-	-	-
**15**	-	-	hAPLN (55)	hAPLN (58)	-	+ (58)
**16**	V (56)	-	hAPLN (62)	-	-	+ (66)

*Class II, III, IV and V refers to either the World Health Organization [16] or International Society of Nephrology/Renal Pathology Society [17] classification criteria of lupus nephritis, histopathological antiphospholipid-associated nephropathy (hAPLN) and end-stage renal disease (ESRD).

### Histopathology

We extracted the histopathological findings available in biopsy reports from all patients. Compared to the LN group, hAPLN patients were associated with occurrence of hypertensive vascular changes (*P* = 0.01), inflammatory infiltrates (*P* = 0.007), interstitial fibrosis (*P* = 0.02), intima changes (*P* < 0.0001), and tubular atrophy (*P* = 0.002).

We found that all hAPLN biopsies without LN findings (n = 10) had inflammatory changes. In contrast, of the patients with hAPLN and concomitant LN 5 had inflammatory changes while the remaining 5 had no signs of inflammation (data not shown).

The histopathological findings are shown in Table 3.

**Table 3:** **Histopathological findings in the study cohort**

	**All patients**	**LN**	**hAPLN**	***P*** **value**
	**(n = 112)**	**(n = 96)**	**(n = 16)**	
Hypertensive vascular changes	37 (33)	29 (30)	8 (50)	0.01
Inflammatory infiltrates	41 (37)	30 (31)	11 (69)	0.007
Interstitial fibrosis	82 (73)	67 (70)	15 (94)	0.02
Intima changes	50 (45)	35 (37)	15 (94)	<0.0001
Tubular atrophy	63 (56)	48 (50)	15 (94)	0.002

Values are shown as n (%). *P* values are comparisons between the two diagnostic groups: lupus nephritis (LN) and histopathological antiphospholipid-associated nephropathy (hAPLN).

### Routine laboratories

In all 112 patients, the median serum creatinine was 77 μmol/L (mean 98, range 39 to 514) at first study biopsy. Serum creatinine levels were higher in the hAPLN group (median 116) as compared to the LN group (median 75; *P* < 0.0001). Albumin and haemoglobin levels did not differ between the hAPLN and LN groups as shown in Table 1.

### Blood pressure

Twenty-seven patients (24.2%) had a diagnosis of, and received treatment for, hypertension at first study biopsy. Of these, nine also had vascular changes consistent with hAPLN. There was a positive association between hAPLN and hypertension (OR = 3.8; 95% CI, 1.2 to 12.3; *P* = 0.03).

The median systolic blood pressure in all patients was 125 mmHg (range 90 to 190 mmHg) and the median diastolic blood pressure at biopsy was 80 mmHg (range 55 to 110 mmHg). Systolic blood pressure was higher in hAPLN patients (median 137 mmHg, range 100 to 190 mmHg) as compared to LN patients (median 120 mmHg, range 90 to 190 mmHg; *P* = 0.01), but no difference was found when comparing diastolic blood pressure. Results are shown in Table 1.

### Antiphospholipid antibodies and lupus anticoagulant

In all patients, 58 (52%) tested positive for aCL, 23 (21%) for anti-β_2_GP1 and 28 (25%) for LA at and/or prior to first study biopsy.

Of the patients in the LN group, 46 (48%) tested positive for aCL, 17 (18%) for anti-β_2_GP1 and 21 (22%) for LA.

Among the hAPLN patients, 12 (75%) tested positive for aCL, 6 (38%) for anti-β_2_GP1 and 7 (44%) for LA. Data on antibodies and LA are presented in Table 1.

hAPLN was positively associated with the presence of aCL (OR = 3.3; 95% CI, 1.0 to 10.8; *P* = 0.05). Although the association between hAPLN and anti-β_2_GP1 or LA (*P* = 0.07 and *P* = 0.06, respectively) did not reach statistical significance, the combinations between all three tests (triple positivity, as defined by Pengo and colleagues [19]) was increased in the hAPLN patients compared to the LN group (OR = 4.2; 95% CI, 1.29 to 13.65; *P* = 0.02).

### Treatment at time of first renal biopsy

Oral prednisolone was prescribed to 76 (67.8%) patients. The median dosage in all patients at the time of biopsy was 8 mg/day (mean 11 mg/day, range 0 to 60 mg/day). The median dosage in the hAPLN group was 15 mg/day and in the LN group was 8 mg/day (*P* = 0.006). The number of patients receiving angiotensin converting enzyme inhibitors and/or angiotensin receptor blockers, acetylsalicylic acid, and warfarin are shown in Table 1.

### Outcome at last follow-up

Development of ESRD was seen in 15 patients, comprising 9/96 (9.4%) in the LN group and 6/16 (37.5%) in the hAPLN group (OR = 5.8; 95% CI, 1.7 to 19.7; *P* = 0.008). The mean time to development of ESRD was 4.4 years following first study biopsy (3.4 years for LN, and 5.9 years for hAPLN).

### HLA-DRB1 allotypes

Patients with hAPLN were more likely to carry the HLA-DRB1*13 allele in comparison to LN patients (OR = 5.1; 95% CI, 1.7 to 15.4; *P* = 0.03 after Bon Ferroni correction). There were no differences between hAPLN and LN for HLA-DRB1*01, *03, *04, *07, *08, *09, *10, *11, *12, *14, *15 or *16 allotypes. Results shown in Table 4.

**Table 4:** **HLA-DRB1 alleles and association with hAPLN**

**HLA-DRB1**	**All patients**	**LN**	**hAPLN**	***P*** **value (after Bonferroni adjustment)**	***P*** **value**
	(n = 110*)	**(n = 94)**	**(n = 16)**		
*01	16 (15)	16 (17)	0 (0)	NS	0.1
*03	43 (39)	36 (38)	7 (44)	NS	0.7
*04	23 (21)	20 (21)	3 (19)	NS	NS
*07	12 (11)	11 (12)	1 (6)	NS	NS
*08	7 (6)	6 (6)	1 (6)	NS	NS
*09	4 (4)	4 (4)	0 (0)	NS	NS
*10	3 (3)	2 (2)	1 (6)	NS	0.4
*11	11 (10)	11 (12)	0 (0)	NS	0.4
*12	4 (4)	4 (4)	0 (0)	NS	NS
*13	28 (26)	19 (20)	9 (56)	0.03	0.002
*14	4 (4)	3 (3)	1 (6)	NS	0.5
*15	49 (45)	44 (47)	5 (31)	NS	0.3
*16	3 (3)	2 (2)	1 (6)	NS	0.4

Values are shown as n (%). *P* values are comparisons between the two diagnostic groups: lupus nephritis (LN) and histopathological antiphospholipid-associated nephropathy (hAPLN). NS, not significant. *Genetic analysis had not been performed on two of the patients.

## Discussion

We detected renal microvascular changes in accordance with hAPLN in 14.3% of the patients in a large cohort of SLE patients with biopsy-proven renal involvement. Overall, hAPLN patients presented with more hypertension, severe histopathology and impaired renal function when compared to patients with pure LN. hAPLN was associated with aCL and with triple aPL positivity. Furthermore, the HLA-DRB1*13 allele was associated with hAPLN in the investigated patients. Our results thus indicate for the first time that a genetic predisposition may contribute to hAPLN in SLE.

In previous studies, at least one histological lesion compatible with hAPLN, either as isolated findings or in addition to LN, was seen in 24 to 32% of patients with SLE [6,7]. Zheng and colleagues [5] detected glomerular microthrombi in 20% of LN patients, which was associated with poorer renal function as well as more severe renal tissue injury. In our study, the observed prevalence of hAPLN was somewhat lower compared to previous studies. Differences in definitions, varying study populations, and indication for renal biopsy are likely to contribute to the observed differences in prevalence. Of note, our study included mainly Caucasian patients as opposed to the Asian population included in previous studies [5,7] and ethical differences could also have contributed.

It has previously been proposed that outer folded or wrinkled basement membrane may be characteristic of microangiopathy in association with APS [22]. However, in the hAPLN group this was only detected in one of the patients (data not shown). Thus, electron microscopy evaluation did not seem to add diagnostic value.

We found a higher proportion of inflammatory infiltrates in the hAPLN group compared to the LN group. In cases with concomitant LN and hAPLN, it was not possible to determine whether the inflammatory infiltrates were caused by either condition. The striking finding of inflammation in the pure hAPLN group, however, strongly indicates that circulatory causes (for example, ischaemia caused by hAPLN or hypertension) have contributed to occurrence of the inflammatory infiltrates.

The overall lower prevalence (8.5%) of hAPLN in all biopsies could also be due to the large number of repeated biopsies in the study cohort. However, the repeated biopsies available gave us a unique opportunity to study the natural history of hAPLN, its development and disappearance. Of note is that, in three of the patients with hAPLN, all findings had disappeared at re-biopsy. Interestingly, this implies that hAPLN can be transient, or possibly focal. Another important observation is that 56% of hAPLN appeared *de novo* in patients who had previously had pure LN. Taken together, these observations demonstrate that repeated biopsies are important when the clinical course indicates an unexplained deterioration of renal function, especially in patients who are aPL positive.

We found that hAPLN was associated with aCL, but a strong trend between hAPLN and anti-β_2_GP1 or LA was also detected. The lack of statistical significance for anti-β2gGP1 and LA was most likely due to the sample size. Consistent with our findings, Tektonidou and colleagues [4] reported an association between APLN and aCL, but also with LA. On the other hand Daugas and colleagues [6] demonstrated a strong association between APLN and LA, but not between APLN and aCL. In a study on occurrence of glomerular microthrombi, there was a strong association with LA and anti-β_2_GP1, but not with aCL [5]. The divergent aPL associations previously reported are likely to be due to methodological issues, as different assays for aPL and LA detection have been used and cut-off values have varied between studies.

The strong association of hAPLN with aCL is consistent with APLN being a manifestation of APS [3]. It is, however, also important to note that 25% of patients with renal findings in accordance with hAPLN had no detectable aPL at all using the assays available at the time. Similar findings have previously been reported [4,7].

It has previously been shown that patients suffering from SLE and systemic hypertension have a worse prognosis concerning renal survival as compared to normotensive individuals [23]. Furthermore, we have previously demonstrated that aPL in SLE patients is a risk factor for cardiovascular disease [24] and both aPL and loss of renal function predicted mortality in SLE [25]. In the present study there was a clear association between hAPLN and hypertension. The hAPLN group was also associated with higher creatinine levels, which is in line with previous publications [4-6]. In addition, we could demonstrate that hAPLN was associated with more severe histopathological renal findings as well as with development of ESRD in long-term follow-up. Considering both previous findings and the present results, there are strong indications that patients with hAPLN belong to a high-risk SLE sub-group. Adequate follow-up and antihypertensive therapy are likely to prevent impairment of renal function, but this should ideally be studied in prospective studies. As a first step towards the performance of prospective treatment studies on patients with APLN, it is also important to reach an international consensus regarding the definition of APLN.

Associations between aPL and the HLA-DRB1*04 allele were reported be Galeazzi and colleagues [13]. We recently demonstrated that the HLA-DRB1*04 and HLA-DRB1*13 alleles alone and in particular the combination of these two alleles (HLA-DRB1*04/*13) were associated both with aPL of several specificities and with an increased risk of macrovascular events among SLE patients [12]. In this study, we extend these observations by demonstrating that microvascular lesions in the kidney are also associated with HLA-DRB1*13. Thus, a similar genetic factor may confer risk of aPL-associated disease in SLE.

The large consecutive sample size, also including repeated biopsies, gave us a unique opportunity to study the prevalence and natural history of hAPLN in SLE patients. Due to retrospective data collection, and lack of repeated measurements of aPL in some cases, we were, however, not able to fully define occurrence of APS.

Somewhat surprisingly, hAPLN patients had higher doses of prednisone at biopsy compared to LN individuals. This could be explained by the more severe phenotype with higher creatinine thus requiring more intense treatment, but the high steroid usage could also have contributed to the more pronounced hypertension seen. However, the more extensive use of corticosteroids, which is unlikely to affect hAPLN findings in general, point to the importance of performing renal biopsies in order to optimize treatment strategies and avoid overtreatment with corticosteroids and immunosuppressants.

Currently there are no consensus guidelines for treatment of APLN, but it is generally recommended that patients should receive anti-hypertensive and anticoagulative treatments. Considering the associated risk for impaired renal function, randomized trials are urgently needed to explore optimal treatment regimens in order to preserve renal function in this sub-group of SLE patients.

## Conclusion

In conclusion, our results demonstrate that hAPLN is a severe, but not uncommon, finding in lupus patients with renal involvement. The association between renal pathology and occurrence of aPL indicates that hAPLN is a separate renal disease entity with different underlying pathogenic mechanisms compared to LN. Due to the increased risk of renal impairment, vasculopathies in LN require an increased awareness. Genetic findings may contribute to hAPLN.

## Abbreviations

aCL: anticardiolipin antibodies
aPL: antiphospholipid antibodies
APLN: antiphospholipid-associated nephropathy
APS: antiphospholipid syndrome
β_2_GP1: β_2_-glycoprotein-1
CI: confidence interval
ELISA: enzyme-linked immunosorbent assay
ESRD: end-stage renal disease
hALPN: histopathological antiphospholipid-associated nephropathy
HLA: human leukocyte antigen
Ig: immunoglobulin
LA: lupus anticoagulant
LN: lupus nephritis
OR: odds ratio
SLE: systemic lupus erythematosus
STAT4: signal transducer and activator of transcription factor 4
TMA: thrombotic microangiopathy
